# Stimulatory effect of icariin on the proliferation of neural stem cells from rat hippocampus

**DOI:** 10.1186/s12906-018-2095-y

**Published:** 2018-01-29

**Authors:** Xiaolong Fu, Shujun Li, Shaoyu Zhou, Qin Wu, Feng Jin, Jingshan Shi

**Affiliations:** 1 Joint International Research Laboratory of Ethnomedicine, Zunyi Medical College, Zunyi, Guizhou China; 2 Key Laboratory of Basic Pharmacology of Ministry of Education, Zunyi Medical College, Zunyi, Guizhou China; 3 Department of Plastic Surgery, Affiliated Hospital of Zunyi Medical College, Zunyi, Guizhou China

**Keywords:** Icariin, Neural stem cells, Proliferation, Cyclin D1, p21

## Abstract

**Background:**

Icariin (ICA), a major ingredient of *Epimediumbrevicornum*, has various pharmacological activities including central nervous system protective functions such as the improvement of learning and memory function in mice models of Alzheimer’s disease. It has been reported that ICA can promote regeneration of peripheral nerve and functional recovery. The purpose of this study was to investigate the potentiating effect of ICA on the proliferation of rat hippocampal neural stem cells, and explore the possible mechanism involved.

**Methods:**

Primary neural stem cells were prepared from the hippocampus of newly born SD rats, and cells were cultured in special stem cell culture medium. Neural stem cells were confirmed by immunofluorescence detection of nestin, NSE and GFAP expression. The effect of ICA on the growth and proliferation of the neural stem cells was evaluated by 5-ethynyl-2-deoxyuridine (EdU) labeling of proliferating cells, and photomicrographic images of the cultured neural stem cells. Further, the mechanism of ICA-induced cell proliferation of neural stem cells was investigated by analyzing the gene and protein expression of cell cycle related genes cyclin D1 and p21.

**Results:**

The present study showed that icariin promotes the growth and proliferation of neural stem cells from rat hippocampus in a dose-dependent manner. Incubation of cells with icariin resulted in significant increase in the number of stem cell spheres as well as the increased incorporation of EdU when compared with cells exposed to control vehicle. In addition, it was found that icariin-induced effect on neural stem cells is associated with increased mRNA and protein expression of cell cycle genes cyclin D1 and p21.

**Conclusions:**

This study evidently demonstrates the potentiating effect of ICA on neural stem cell growth and proliferation, which might be mediated through regulation of cell cycle gene and protein expression promoting cell cycle progression.

**Electronic supplementary material:**

The online version of this article (10.1186/s12906-018-2095-y) contains supplementary material, which is available to authorized users.

## Background

Alzheimer’s disease (AD) is a chronic neurodegenerative disorder and the most prevalent cause of dementia with ageing. It was estimated that there were approximately 29.8 million people affected by this neurodegenerative disorder worldwide in 2015 [1]. Despite extensive research, the cause of pathological progression of the AD remains unknown. There is currently no cure for AD. Pharmacological treatment of AD such as acetylcholinesterase inhibitors and NMDA channel blocker has some beneficial effects on cognitive and behavioral symptoms, but it does not stop or reverse the progression of the disease [2]. Discovering an effective therapeutic drug to treat AD patients continues to be a challenge. One of the core features of AD is the dysfunction or loss of the neural cells in hippocampus resulting in the functional impairment. In recent years, it has been an active research area to replace neural tissue loss through transplantation of neural stem cells (NSCs) for the treatment of AD [3]. NSCs can be cultured in vitro as neurospheres that are composed of neural stems cells and progenitors. The NSCs can grow and multiply in vitro medium, and then be activated to differentiate into different nerve cells to be transplanted within the brain to replace damaged cells. In this regard, the growth and proliferation of the neural stem cells in vitro are critical as the number of cells available for transplantation is essential to achieve beneficial effect in replacing injury or loss of the neuronal cells.

It has been well established that many growth factors such as epidermal growth factor (EGF), fibroblast growth factor (FGF), platelet-derived growth factor, and granulocyte-colony stimulating factor (G-CSF) stimulate the proliferation of NSCs [4, 5]. Exploring the effect of pharmacological manipulation, such as usage of traditional medicine, on the growth and proliferation of neural stem cells is thus valuable towards the development of the stem cells therapies of the neurodegenerative disorders.

Icariin is an active compound of the total flavonoid extracted from the traditional Chinese herb Epimedium brevicornum Maxim. A number of studies report that icariin has a variety of pharmacological functions including neuroprotective effects [6, 7]. Previous studies in our laboratory showed that ICA can protect rat brain dysfunction caused by lipopolysaccharide, and improve spatial learning and memory of AD mice induced by Aβ_25–35_ [8, 9]. We also showed that ICA can attenuate D-galactose induced neurodegeneration and behavioral disorder through up-regulating hippocampal brain-derived neurotrophic factor (BDNF), and tropomyosin receptor kinase mRNA and protein expression [10]. It has been reported that ICA stimulates the proliferation and differentiation of human bone mesenchymal stem cells promoting bone formation [11, 12]. Recent studies demonstrated that local administration of icariin inhibits neuron apoptosis and promote the regeneration of peripheral nerve and functional recovery [13, 14]. However, whether ICA can regenerate neuronal cells and improve the function of neurodegenerative disorders through potentiating the proliferation and differentiation of neural stem cells remains unknown. The objective of the present study was to investigate the effect of ICA on the proliferation and underlying mechanism.

## Methods

### Experimental animals

Healthy SPF Sprague-Dawley rats (220 to 260 g) were obtained from the Experimental Animal Center of Daping Hospital, Research Institute of Surgery, Third Military Medical University (SPFgrade, Certificate NO. SCXK 2012–0005). The animals were maintained under a 12 h light/dark cycle in temperature (23 ± 1 °C) and humidity (relative, 60%)-controlled rooms and allowed free access to food and water. All animal experiments were strictly carried out in accordance with NIH guidelines for the Care and Use of Laboratory Animals (NIH Publications No. 80–23, revised 1996), and the study protocol was approved by the Animal Experimentation Ethics Committee of Zunyi Medical University.

### Chemical reagents

Icariin (ICA, purity of greater than 98.6% by high-performance liquid chromatography) was purchased from Nanjing Zelang Medical Technology Corporation Ltd. (Nanjing, China), and stock solution of ICA was prepared in dimethyl sulfoxide (DMSO). SD rat neural stem cell culture medium, and Serum-free 6-well culture plates were obtained from Cyagen Biosciences Inc. (Guangzhou, China). The Cell-Light EdU imaging detection kit was purchased from RiboBio Co., Ltd. (Guangzhou, China).

### Isolation and culture of neural stem cells

Primary neural stem cells were prepared from newly born SD rats. The rats were decapitated and their brains were removed and dissected in a petri plate containing calcium and magnesium-free Hank’s balanced salt solution (HBSS; Solarbio, Beijing, China) with 10 mM Hepes and 1% streptomycin–penicillin. For the preparation of hippocampal stem cells, the hippocampi (*n* = 15 for each experiment) were dissected free of the cortices under a dissecting microscope and washed thoroughly in DMEM/F12 medium (Thermo, Shanghai, China). Hippocampi were then triturated 30–35 times in 3 mL of DMEM/F12 medium using a fire-polished pipet, and the cell suspension was filtered through a 70 μm cell strainer (BD Biosciences) in 10 mL of DMEM/F12 medium. The cells were centrifuged at 400 × *g* for 5 min and the pellet was resuspended in 1 mL of stem cell culture medium comprised of SD Rat Neural Stem Cell Growth Medium (Cyagen Biosciences) supplemented with 2% B27, 1% glutamine, 0.2 mg/mL recombinant murine epidermal growth factor, 0.1 mg/mL recombinant human fibroblast growth factor-2, and 5 mg/mL heparin. The resultant cells were counted and plated in 6-well plates at a density of 1 × 10^6^/mL in each well. The medium was changed every 48 h. Neurospheres were passaged at a density of 5 × 10^5^/mL when they were around 100 μm in diameter. Cultures were monitored daily. For experiments involving neuronal differentiation, neurospheres were dissociated into single cells in accutase (Sigma-Aldrich, St. Louis, USA), then plated at a density of 50,000 cells per coverslip in 24-well plates coated with poly-L-lysine (Sigma-Aldrich, St. Louis, USA). NSCs were incubated in neurogenic differentiation medium (Cyagen Biosciences). The medium was replaced half every 48 h.

### Immunofluorescence and imaging

Primary neural stem cells were detected and confirmed via immunofluorescence. Cultured cells were washed three times with PBS (pH 7.4) and fixed in 4% paraformaldehyde for 30 min. Cells were then permeabilized by incubation in 0.1% Triton X-100 in PBS solution. Cells were blocked with 5% goat serum for 30 min, then incubated overnight at 4 °C with primary antibodies. Antibodies used included mouse anti-Nestin (1: 500, Abcam Co., Ltd., Cambridge, UK), rabbit anti-NSE (1: 200, Abcam Co., Ltd., Cambridge, UK) and mouse anti-GFAP (1: 1000, Abcam Co., Ltd., Cambridge, UK). In the following day, cells were incubated with appropriate species-specific fluoro-conjugated secondary antibodies (Alexa Fluor 488-labeled goat anti-rabbit, goat anti-mouse and 594-labeled goat anti-rabbit IgG (H + L), 1: 1000, Sigma-Aldrich, St. Louis, MO, USA) for 2 h. Meanwhile, the nucleus was stained with DAPI (Sigma-Aldrich, St. Louis, USA). Stained cells were mounted and examined by IX 73 fluorescence microscopy (OLYMPUS). Images were acquired using cellSens Dimension software.

### 5-ethynyl-2-deoxyuridine (EdU) proliferation assay

The incorporation of 5-ethynyl-2-deoxyuridine (EdU) has been used to detect DNA synthesis in proliferating neural cells as described previously [15, 16]. Primary neural stem cells which had been grown for 48 h were incubated with 10 μM EdU for 24 h, blown into single cells, fixed in 4% paraformaldehyde, permeabilized by 0.5% Triton X-100, and stained with EdU according to the manufacturer’s protocol and DAPI for cell nuclei. Finally, the stained cells were imaged under the fluorescent microscope (OLYMPUS).

### The effect of ICA on neural stem cell growth and proliferation

In order to determine whether ICA affects neural stem cells growth and proliferation, the primary culture of the neural stem cells was exposed to different concentrations of ICA. The cells were cultured in 6-well plates at a cell density of 1 × 10^6^ cells per well. Three days following preparation of the neural stem cells, cells were treated with ICA (0, 50, and 100 μM, respectively). At day 7 of the culture, the neural stem cells were blown into single cells by repeated pipetting with a fine pipet, and in vitro proliferation of the cells were determined by counting through a cell counter (CountersterKIC1000, Shanghai, China). Cell proliferation was also evaluated by quantifying growing cells’ incorporation of 5-ethynyl-2-deoxyuridine (EdU). ICA was added to the cultured cells at different concentrations (0, 50, and 100 μM, respectively) after three days of culture. Following 12 h incubation of cells with ICA, EdU was added to the cultured cells. After 24 h, cells were collected to assess the effect of ICA on cell proliferation by examining the immunofluorescence of EdU.

### The effect of ICA on gene and protein expression of cyclin D1 and p21

Gene expression of both cyclin D1 and p21 was determined by Quantitative Real-Time PCR (qRT-PCR) using the SYBR green PCR Master Mix (TaKaRa Biotechnology Co. Ltd., Dalian, China). Total RNA from the cultured cells was isolated by Trizol reagent (TaKaRa Biotechnology Co. Ltd., Dalian, China) and purified with RNeasy Kits (TaKaRa Biotechnology Co. Ltd., Dalian, China). The primer sequences for the selected genes were designed with the Primer 3 software and listed in Table 1. Total RNA was reversely transcribed by MuLV reverse transcriptase and Oligo-dT primers (TaKaRa Biotechnology Co. Ltd., Dalian, China). The SYBR green PCR Master Mix (TaKaRa Biotechnology Co. Ltd., Dalian, China) was used for real-time PCR analysis. The relative differences in expression between groups were reflected as cycle time (Ct) values normalized with β-actin of the same sample. The relative mRNA expression levels in each group were calculated and expressed as a percentage relative to sham group as 100%.

**Table 1:** Primer sequences for gene expression by real-time PCR

Gene	GenBank Accession#	Forward	Reverse
cyclin D1	X75207	CACAACGCACTTTCTTTCCA	CTTGGGATCGATGTTCTGCT
p21	U09793	CTGGTGATGTCCGACCTGTTC	CTGCTCAGTGGCGAAGTCAAA
β-action	NM031144	GGAGATTACTGCCCTGGCTCTTA	GACTCATCGTACTCCTGCTTGCTG

The protein expression of cyclin D1 was analyzed by Western blot. The cultured cells were homogenized rapidly at 4 °C in the RIPA lysis buffer (150 mM NaCl, 0.5% deoxycholate, 1% NP-40, 0.1% sodium dodecyl sulfate, 2 mM phenylmethylsulfonyl fluoride, and 50 mM Trishydrochloric acid, pH 7.4). Protein concentrations were determined using an enhanced BCA protein assay kit (Proteintech Group Inc., Wuhan, China) separated by SDS-polyacrylamide gel electrophoresis (SDS-PAGE) and transferred onto a PVDF membrane. The membrane was incubated with 5% non-fat dry milk in Tris-buffered saline with Tween (TBST) (10 mM Tris, 150 mM NaCl, 0.05% Tween-20, pH 7.5) for 2 h at room temperature with constant shaking, and then probed with corresponding primary antibodies against cyclin D1 (1: 100, Abcam Co., Ltd., Cambridge, UK), p21 (1: 1000, Abcam Co., Ltd., Cambridge, UK), and β-actin (1: 5000, Proteintech Group Inc., Wuhan, China) at 4 °C overnight. After incubation with appropriate horseradish peroxidase-coupled secondary antibodies for 2 h at room temperature, immunoreactive proteins were visualized using the enhanced ECL Western blot detection kit (Beyotime, Shanghai, China) and exposed to Gel Imaging (BioRad, Shanghai, China).

### Data analysis

All results were presented as mean ± SD of at least three individual experiments. One-way analysis of variance (ANOVA) and t-test were used to determine statistical differences between the groups. All analyses were carried out using SPSS 17.0 software. A value of *P* < 0.05 was considered significant difference.

## Results

### Cellular morphological examination of neural stem cells

**Fig. 1:**
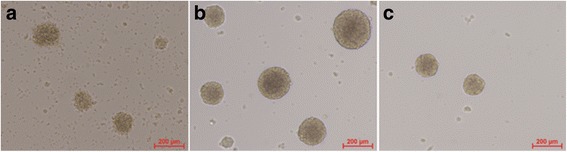
Morphological characteristics of neural stem cells cultured at different time points. Isolated neural cells were cultured in neural stem cells medium, and morphological changes of the cells were observed under a microscopy. **a** Primary culture of neural stem cells at day 3. **b** Primary culture of neural stem cells at day 7. **c** Subculture of the day 7

The cellular morphology of neural stem cells was examined by microscopy for changes in appearance during each passage. Neural stem cells are capable of forming in vitro into neurospheres, which possess three fundamental characteristics: proliferation, self-renewal, and multipotency [17]. It was observed that new floating neurospheres containing varying number of cells were formed at day 3 of the culture as a consequence of cell growth and proliferation. These cellular neurospheres were identified as well-defined spherical cell clusters as well as more irregular cell clusters (Fig. 1). Cilia were also present on the surface or outer edges of some of the neurospheres, indicating that the neurospheres were in a healthy condition. The neurospheres were found to be capable of self-renewal while in the culture of neural stem cell medium.

### Confirmation of NSC by immunofluorescent staining of NSC markers

**Fig. 2:**
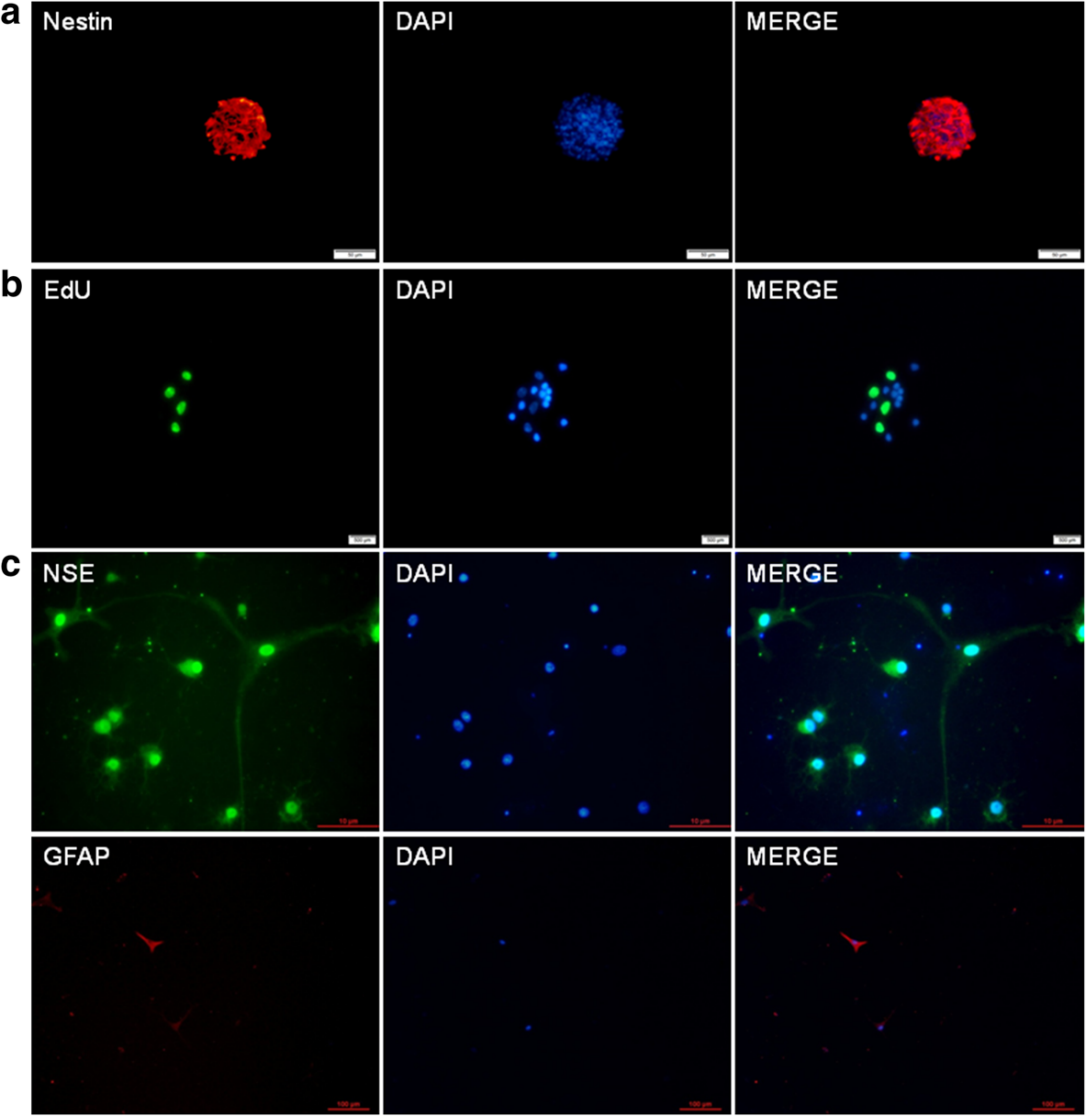
Immunofluorescent staining of NSCs derived from rat hippocampus. **a** Nestin immunoreactivity (red) was positive. **b** EdU immunoreactivity (green) was positive compared to control. **c** The immunofluorescence staining showed positive NSE (green) and GFAP (red) expression. DAPI (blue) was used to stain the nuclei

NSCs are confirmed by examining the protein expression of nestin, a type VI intermediate filament protein, which is considered as a molecular marker of neural stem cells [18]. Immunocytochemical staining of NSCs showed that neurosphere isolates from all the experimental groups were positively stained for nestin at passage 3 of the culture of primary cells (Fig. 2a). Next, we determined the proliferative activity of NSCs by examining the incorporation of EdU into the cultured neurospheres. Immunofluorescent analysis of the cells revealed a robust increase in fluorescence intensity in cells exposed to EdU in relative to the control cells, indicating the proliferation of the stem cells (Fig. 2b). Finally, we explored the capacity of differentiation of the NSCs. The neurospheres were induced to differentiate into neurons and glial cells by growing in a specific differentiation medium. After induction and growth for 1 week, the differentiated cells were examined for the expression of NSE, a marker for nerve cells, and GFAP, a marker for glial cells. As shown in Fig. 2c, the expression of both NSE and GFAP were positive (Fig. 2c). These results verified the differentiation capacity of the NSCs derived from the differentiated neurospheres.

### The effect of ICA on the growth and proliferation of NSCs

**Fig. 3:**
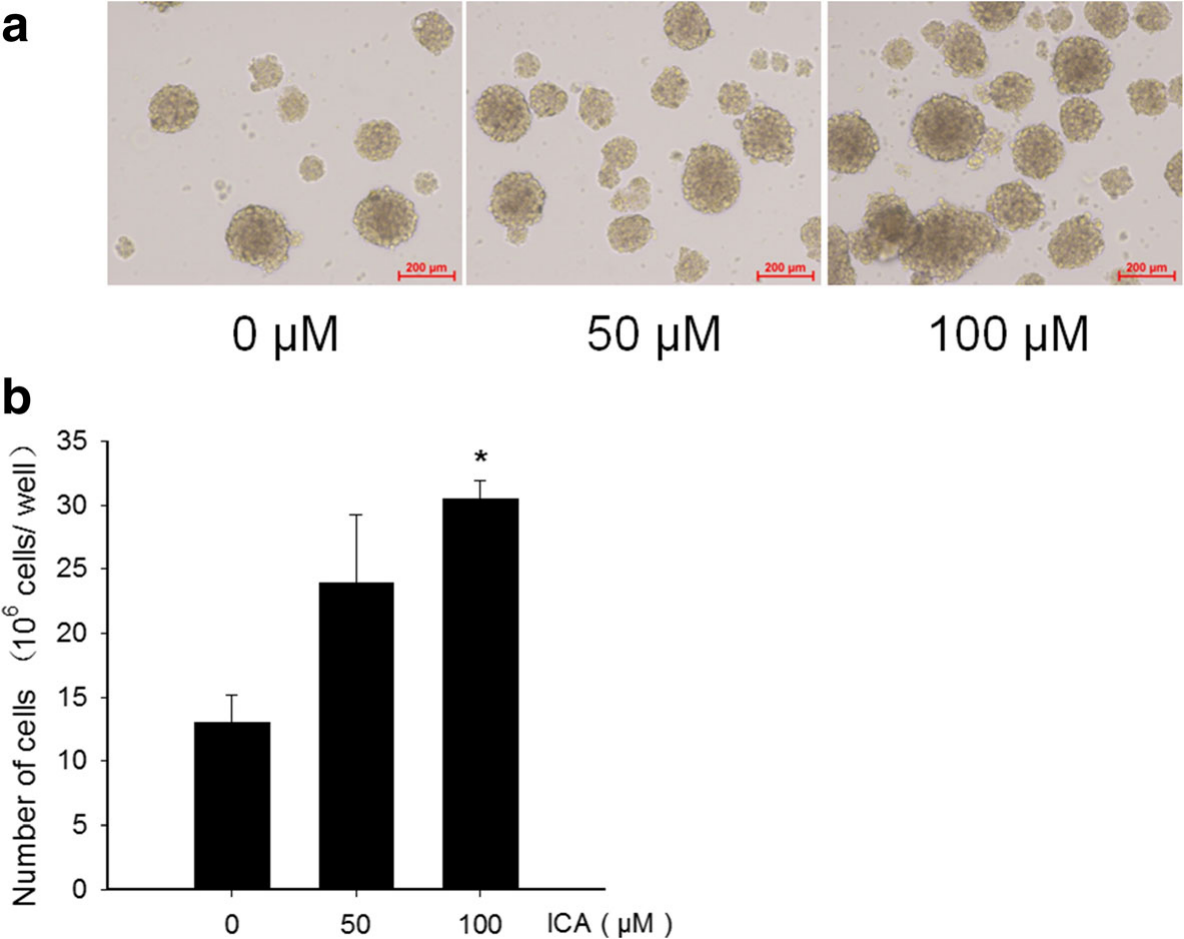
Effect of ICA on the proliferation of NSCs. **a** Photomicrographic images were representative of control, 50 and 100 μM ICA-treated rat hippocampal cultures. **b** Quantitation of NSCs under different concentrations of ICA treatment. Data are from three independent experiments, and the results are plotted as mean ± SD of NSCs. ^*^P < 0.01 vs control

**Fig. 4:**
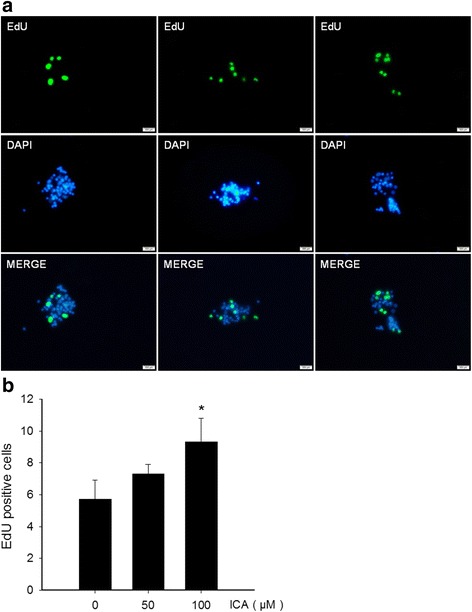
Proliferating cells were examined using the EdU assay. ICA increased the EdU-positive cells. **a** EdU incorporation visualized by specific EdU antibody immunofluorescence. Photomicrographic images were representative of control, 50 and 100 μM ICA-treated rat hippocampal cultures. **b** Quantitation of EdU-positive cells from 10 randomly selected fields per slide. Data are from three independent experiments, and the results are presented as mean ± SD of three individual experiments. ^*^P < 0.05 vs control

To determine whether ICA is able to potentiate the growth and proliferation, the primary NSCs at day 3 were exposed to different concentrations of ICA (0, 50, 100 μM, respectively). The NSCs continued to grow to day 7, and then were blown into single cells by repeated pipetting with a fine pipette, then subjecting to cell counting. As shown in Fig. 3, cells exposed to 50 and 100 μM ICA gave rise to 19.0 ± 7.6 × 10^6^ and 30.6 ± 2.6 × 10^6^ cells/well, respectively, compared with 9.8 ± 0.8 × 10^6^ cells/well observed in the control group, indicating that ICA increased the number of NSCs in a concentration dependent manner (Fig. 3a-b). Further, we performed EdU incorporation experiments to verify the proliferative effect of ICA. Hippocampal NSC cultures were treated with 50 and 100 μM ICA, respectively. Each set of cultures were exposed to EdU (10 μM). It appeared that cells treated with ICA had a significantly greater number of EdU-positive cells relative to the control (Fig. 4a-b).

### Effect of ICA on the expression of cyclin D1 and p21 in NSCs

**Fig. 5:**
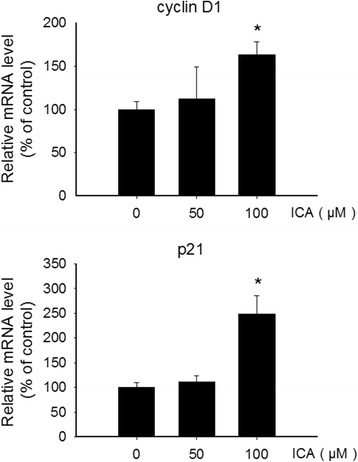
Effect of ICA on the expression of cyclin D1 and p21 mRNA in NSCs. The expression of cyclin D1 and p21 mRNA was detected by qRT-PCR. Values represent mean ± SD of five individual experiments. ^*^P < 0.01 vs Control

**Fig. 6:**
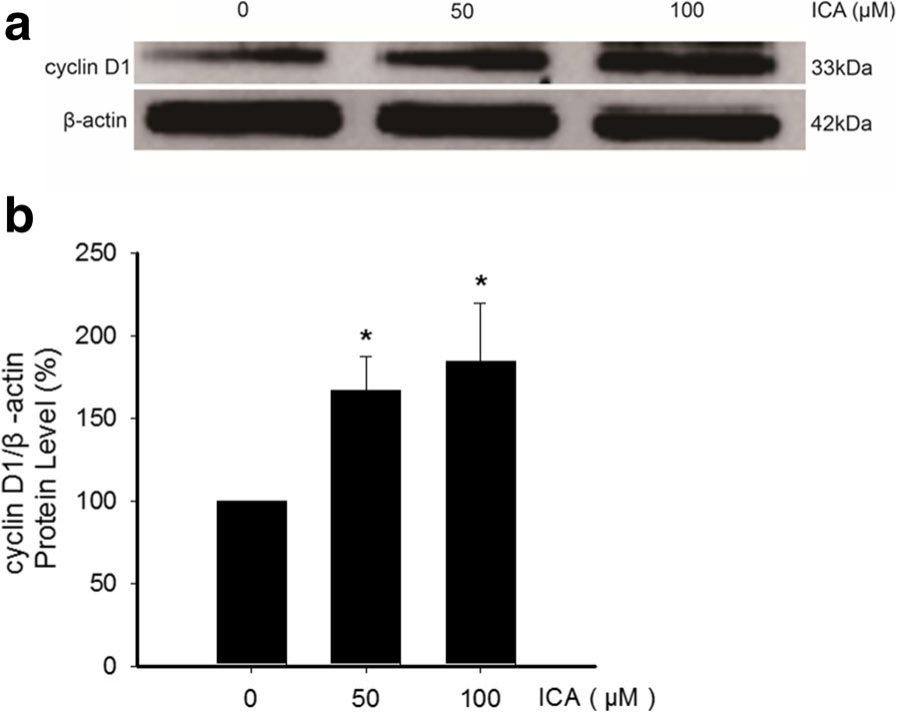
Effect of ICA on cyclin D1 protein level in NSCs. The expression of cyclin D1 protein was determined by Western blot, and β-actin was used as loading control. **a** Representative blotting of cyclin D1 protein expression. **b** Quantitation of cyclin D1 protein level. Values represent mean ± SD of five individual experiments. ^*^P < 0.01 vs Control

To explore the mechanisms underlying the effect of ICA on cell proliferation, the expression of mRNA of both cyclin D1 and p21 was detected by quantitative RT-PCR, while the protein expression of cyclin D1 was examined by Western blot. ICA at 100 μM significantly increased the expression levels of cyclin D1 and p21 mRNA, which were significantly higher than the control group (Fig. 5). Consistent with the mRNA expression, the higher protein expression of cyclin D1 was found in cells exposed to 50 and 100 μM ICA, when compared with control cell (Fig. 6). These data demonstrated that ICA is able to promote proliferation of hippocampal stem cells in vitro.

## Discussion

Neural stem cells are under intensive studies for use in cell-based therapies because their differentiation abilities, which may hold a potential for the cure of neurodegenerative diseases such as AD. In the present study, we demonstrated that ICA is able to promote the growth and proliferation of neural stem cells prepared from rat hippocampus in an in vitro culture system. The in vitro proliferation and differentiation properties of the stem cells are critical as the low proliferation and survival rates of the transplanted stems cells remains to be obstacles to the successful transplantation therapy [19].

Despite the reports that ICA stimulates the proliferation and differentiation of the stem cells in vitro [13, 14], little is known on the effect of ICA on neural stem cells proliferation. In the present study, we prepared neural stem cells from rat hippocampus, and confirmed the properties of the proliferation and differentiation of the neural stem cells, and demonstrated that ICA significantly augments the growth and proliferation of the primary neural stem cells. These results clearly demonstrate ICA’s effect on neural stem cells in vitro, however, whether ICA possesses a similar effect in vivo remains unknown. Previously we have shown that administration of ICA inhibits lipopolysaccharide neurotoxicity and improves the behavioral function of Aβ_25–35_ induced AD mice [8, 9]. It has been demonstrated that neurogenesis occurs in the adult human hippocampus in late 1990’s [20]. Since then, environmental and pharmacological stimulation of neurogenesis in the adult has been an active area of investigation in search for an effective approach to generate replacement cells [21, 22]. Interestingly, it has been reported in recent years that a number of traditional Chinese medicine such as Chinese angelica, ligustrazine, gardenia, herbal astragaloside and ginseng saponins are able to promote neural stem cells proliferation and differentiation [23, 24]. Whether the neuroprotective effect of ICA in vivo pertains to the proliferation and differentiation of neural stem cells, is thus worthy of further investigation.

To understand the molecular mechanism of ICA-mediated proliferation of neural stem cells, we next investigate the regulation of cell cycle of neural stem cells in response to ICA treatment. The cell cycle is a dynamic process, and modulation on any stage of the cell cycle may influence the proliferation of the cell [25]. We therefore investigated the expression levels of cyclin D1 and p21, two important genes in cell cycle regulation. Specifically, we examined the mRNA level of both cyclin D1 and p21, and the protein level of cyclin D1.

Cyclin D1 as a cell cycle protein is a human gene CCND1 encoding protein [26], consisting of 295 amino acids. Under the regulation of the cyclin dependent kinase [27], cyclin D1 plays an important role in normal cell proliferation and growth [28, 29]. Modulation of cyclin D1 has been involved in the mechanism of proliferation of a variety of cell types [30, 31]. We demonstrated that ICA enhances significantly the expression of cyclin D1. Further, we showed that the protein expression of cyclin D1 is accordingly increased in the cells exposed to ICA. These results suggest a role of cyclin D1 in the mechanism of ICA-mediated cell proliferation.

To further understand the molecular mechanism of ICA-stimulated proliferation of neural stem cells, we determined the gene expression of p21, a potent cyclin-dependent kinase inhibitor involved in cell growth and differentiation, which plays an important role in cell physiology and many pathological processes [32–34]. It has been demonstrated that in NSCs and hematopoietic stem cells (HSCs), p21-mediated cell cycle restriction is critical for self-renewal, and p21-deficient NSCs and HSCs display compromised proliferating activity over time [35, 36]. Studies suggest that p21 may regulate NSC self-renewal through repressing Sox2 gene expression [37]. The experimental results in the present study showed that compared with the control group, the gene expression of p21 was markedly increased in the cells exposed to ICA, in a concentration-dependent manner. These results further confirmed the modulation of ICA on cell cycle regulation which may be a mechanism involved in the ICA-mediated effect on cell growth and proliferation of the neural stem cells isolated from rat hippocampus. Despite the effect of ICA on the expression of cyclin D1 and p21, the precise molecular mechanism by which ICA regulates the expression of cyclin D1 and p21 remains unknown. A number of cellular signaling pathways have been described to regulate the expression of cyclin D1 and p21. For instance, it has been recently reported that Dixdc1 (DIX domain containing 1) is capable of promoting Schwann cell proliferation by targeting cyclin D1 and p21 through PI3K activation [38]. Whether PI3K or any other cellular signaling pathways are involved in the ICA-mediated modulation of cyclin D1 and p21 regulation is worthy of further investigation.

## Conclusions

In conclusion, the present study demonstrates that ICA is capable of promoting the growth and proliferation of NSCs, which is related to the ICA-mediated modulation on the regulation of cell cycle genes cyclin D1 and p21. These results suggest a potential of ICA as an alternative treatment of AD disease.

## Additional files

Additional file 1: Table S1. Raw data for Fig. 3. (DOCX 18 kb)

Additional file 2: Table S2. Raw data for Fig. 4. (DOCX 18 kb)

Additional file 3: Table S3. Raw data for Fig. 5. (DOCX 25 kb)

Additional file 4: Table S4. Raw data for Fig. 6. (DOCX 19 kb)

## Abbreviations

AD: Alzheimer’s disease
ANOVA: Analysis of variance
BCA: Bicinchoninic acid
BDNF: Brain-derived neurotrophic factor
DAPI: 4′,6-diamidino-2-phenylindole
DMSO: Dimethyl sulfoxide
EdU: 5-ethynyl-2-deoxyuridine
EGF: Epidermal growth factor
FGF: Fibroblast growth factor
G-CSF: Granulocyte-colony stimulating factor
GFAP: Glial fibrillary acidic protein
ICA: Icariin
NMDA: N-methyl-D-aspartic acid
NSCs: Neural stem cells
NSE: Neuron-specific enolase
qRT-PCR: Quantitative Real-Time PCR
SPSS: Statistical Package for Social Sciences
TBST: Tris-buffered saline with Tween
