# Supplementary material for: Stimulatory effect of icariin on the proliferation of neural stem cells from rat hippocampus
Source: BMC Complement Altern Med. 2018 Jan 29;18:34. doi: 10.1186/s12906-018-2095-y (PMC5789743; doi:10.1186/s12906-018-2095-y)
Supplement: Supplementary file 1 — Raw data for Fig. 3. (DOCX 18 kb) [file 12906_2018_2095_MOESM1_ESM.docx]

**Table S1.** Raw data for Fig. 3.

**Number of neural stem cells（10^6^ cells/ well）**

| Group | Experiment 1 | Experiment 2 | Experiment 3 | Mean | SD |
| --- | --- | --- | --- | --- | --- |
| Control | 9.6 | 10.7 | 9.2 | 9.8 | 0.8 |
| ICA 50 μM | 12.1 | 27.1 | 17.7 | 19.0 | 7.6 |
| ICA 100 μM | 28.6 | 33.5 | 29.6 | 30.6 | 2.6 |
