# Supplementary material for: Stimulatory effect of icariin on the proliferation of neural stem cells from rat hippocampus
Source: BMC Complement Altern Med. 2018 Jan 29;18:34. doi: 10.1186/s12906-018-2095-y (PMC5789743; doi:10.1186/s12906-018-2095-y)
Supplement: Supplementary file 2 — Raw data for Fig. 4. (DOCX 18 kb) [file 12906_2018_2095_MOESM2_ESM.docx]

**Table S2.** Raw data for Fig. 4.

**EdU positive cells**

| Group | Experiment 1 | Experiment 2 | Experiment 3 | Mean | SD |
| --- | --- | --- | --- | --- | --- |
| Control | 5.4 | 4.6 | 7.0 | 5.7 | 1.2 |
| ICA 50 μM | 7.9 | 6.7 | 7.4 | 7.3 | 0.6 |
| ICA 100 μM | 9.2 | 10.9 | 7.9 | 9.3 | 1.5 |
