# Supplementary material for: Stimulatory effect of icariin on the proliferation of neural stem cells from rat hippocampus
Source: BMC Complement Altern Med. 2018 Jan 29;18:34. doi: 10.1186/s12906-018-2095-y (PMC5789743; doi:10.1186/s12906-018-2095-y)
Supplement: Supplementary file 3 — Raw data for Fig. 5. (DOCX 25 kb) [file 12906_2018_2095_MOESM3_ESM.docx]

**Table S3.** Raw data for Fig. 5.

**The values of cyclin D1 and p21 mRNA expression**

|  | **β-actin** |  |  |  |  |  | **cyclin D1** |  |  |  |  |  |  |  |  |
| --- | --- | --- | --- | --- | --- | --- | --- | --- | --- | --- | --- | --- | --- | --- | --- |
| Group | ct1 | ct2 | ct3 | average | dct | 2^(-dct) | ct1 | ct2 | ct3 | average | dct | 2^(-dct) | % of actin | **Meam** | **SD** |
| Control 1 | 16.14 | 16.23 | 16.03 | 16.13 | 0.13 | 0.91 | 18.67 | 18.78 | 18.56 | 18.67 | 2.67 | 0.16 | **17.3** | **17.1** | **1.5** |
| Control 2 | 16.41 | 16.32 | 16.38 | 16.37 | 0.37 | 0.78 | 19.39 | 19.20 | 18.97 | 19.18 | 3.18 | 0.11 | **14.2** |  |  |
| Control 3 | 16.25 | 16.24 | 16.44 | 16.31 | 0.31 | 0.81 | 18.91 | 18.89 | 18.82 | 18.87 | 2.87 | 0.14 | **16.9** |  |  |
| Control 4 | 16.57 | 16.42 | 16.41 | 16.46 | 0.46 | 0.73 | 19.06 | 19.12 | 19.48 | 19.22 | 3.22 | 0.11 | **14.8** |  |  |
| Control 5 | 17.39 | 17.84 | 17.59 | 17.61 | 1.61 | 0.33 | 19.75 | 19.85 | 19.66 | 19.75 | 3.75 | 0.07 | **22.6** |  |  |
| ICA 50 μM 1 | 17.03 | 16.86 | 16.20 | 16.70 | 0.70 | 0.62 | 18.18 | 17.82 | 17.60 | 17.87 | 1.87 | 0.27 | **44.5** | **19.2** | **6.4** |
| ICA 50 μM 2 | 15.65 | 15.52 | 15.61 | 15.59 | -0.41 | 1.32 | 18.09 | 18.11 | 18.37 | 18.19 | 2.19 | 0.22 | **16.5** |  |  |
| ICA 50 μM 3 | 16.21 | 16.25 | 15.86 | 16.10 | 0.10 | 0.93 | 19.55 | 19.62 | 19.15 | 19.44 | 3.44 | 0.09 | **9.9** |  |  |
| ICA 50 μM 4 | 16.94 | 16.66 | 16.73 | 16.78 | 0.78 | 0.58 | 19.84 | 19.45 | 19.36 | 19.55 | 3.55 | 0.09 | **14.6** |  |  |
| ICA 50 μM 5 | 15.49 | 15.62 | 15.54 | 15.55 | -0.45 | 1.37 | 18.80 | 18.94 | 18.65 | 18.79 | 2.79 | 0.14 | **10.6** |  |  |
| ICA 100 μM 1 | 16.38 | 16.62 | 16.80 | 16.60 | 0.60 | 0.66 | 18.32 | 18.49 | 18.68 | 18.50 | 2.50 | 0.18 | **26.9** | **27.9** | **2.6** |
| ICA 100 μM 2 | 17.07 | 16.24 | 15.85 | 16.39 | 0.39 | 0.77 | 17.95 | 17.79 | 17.70 | 17.81 | 1.81 | 0.28 | **37.2** |  |  |
| ICA 100 μM 3 | 16.95 | 16.71 | 16.50 | 16.72 | 0.72 | 0.61 | 18.93 | 18.57 | 18.57 | 18.69 | 2.69 | 0.16 | **25.6** |  |  |
| ICA 100 μM 4 | 17.70 | 17.44 | 17.57 | 17.57 | 1.57 | 0.34 | 19.47 | 19.33 | 19.37 | 19.39 | 3.39 | 0.10 | **28.4** |  |  |
| ICA 100 μM 5 | 18.22 | 18.20 | 18.38 | 18.27 | 2.27 | 0.21 | 20.32 | 20.31 | 20.82 | 20.48 | 4.48 | 0.04 | **21.5** |  |  |

|  | **β-actin** |  |  |  |  |  | **p 21** |  |  |  |  |  |  |  |  |
| --- | --- | --- | --- | --- | --- | --- | --- | --- | --- | --- | --- | --- | --- | --- | --- |
| Group | ct1 | ct2 | ct3 | average | dct | 2^(-dct) | ct1 | ct2 | ct3 | average | dct | 2^(-dct) | % of actin | **Meam** | **SD** |
| Control 1 | 16.14 | 16.23 | 16.03 | 16.13 | 0.13 | 0.91 | 24.37 | 23.82 | 23.55 | 23.91 | 7.91 | 0.00 | **0.46** | **0.71** | **0.07** |
| Control 2 | 16.41 | 16.32 | 16.38 | 16.37 | 0.37 | 0.78 | 23.40 | 23.33 | 23.14 | 23.29 | 7.29 | 0.01 | **0.82** |  |  |
| Control 3 | 16.25 | 16.24 | 16.44 | 16.31 | 0.31 | 0.81 | 23.35 | 23.29 | 22.93 | 23.19 | 7.19 | 0.01 | **0.85** |  |  |
| Control 4 | 16.57 | 16.42 | 16.41 | 16.46 | 0.46 | 0.73 | 23.45 | 23.48 | 23.74 | 23.56 | 7.56 | 0.01 | **0.73** |  |  |
| Control 5 | 17.39 | 17.84 | 17.59 | 17.61 | 1.61 | 0.33 | 24.93 | 24.81 | 24.59 | 24.78 | 8.78 | 0.00 | **0.69** |  |  |
| ICA 50 μM 1 | 17.03 | 16.86 | 16.20 | 16.70 | 0.70 | 0.62 | 23.76 | 23.21 | 22.80 | 23.26 | 7.26 | 0.01 | **1.06** | **0.79** | **0.09** |
| ICA 50 μM 2 | 15.65 | 15.52 | 15.61 | 15.59 | -0.41 | 1.32 | 22.69 | 22.51 | 22.67 | 22.62 | 6.62 | 0.01 | **0.77** |  |  |
| ICA 50 μM 3 | 16.21 | 16.25 | 15.86 | 16.10 | 0.10 | 0.93 | 23.45 | 23.66 | 23.13 | 23.42 | 7.42 | 0.01 | **0.63** |  |  |
| ICA 50 μM 4 | 16.94 | 16.66 | 16.73 | 16.78 | 0.78 | 0.58 | 23.77 | 23.32 | 23.52 | 23.54 | 7.54 | 0.01 | **0.92** |  |  |
| ICA 50 μM 5 | 15.49 | 15.62 | 15.54 | 15.55 | -0.45 | 1.37 | 23.10 | 23.06 | 22.93 | 23.03 | 7.03 | 0.01 | **0.56** |  |  |
| ICA 100 μM 1 | 16.38 | 16.62 | 16.80 | 16.60 | 0.60 | 0.66 | 23.12 | 22.98 | 22.91 | 23.00 | 7.00 | 0.01 | **1.18** | **1.76** | **0.27** |
| ICA 100 μM 2 | 17.07 | 16.24 | 15.85 | 16.39 | 0.39 | 0.77 | 23.01 | 22.45 | 22.23 | 22.56 | 6.56 | 0.01 | **1.38** |  |  |
| ICA 100 μM 3 | 16.95 | 16.71 | 16.50 | 16.72 | 0.72 | 0.61 | 22.85 | 22.46 | 22.36 | 22.56 | 6.56 | 0.01 | **1.75** |  |  |
| ICA 100 μM 4 | 17.70 | 17.44 | 17.57 | 17.57 | 1.57 | 0.34 | 23.60 | 23.32 | 23.28 | 23.40 | 7.40 | 0.01 | **1.76** |  |  |
| ICA 100 μM 5 | 18.22 | 18.20 | 18.38 | 18.27 | 2.27 | 0.21 | 23.44 | 23.35 | 23.58 | 23.45 | 7.45 | 0.01 | **2.74** |  |  |
