# Supplementary material for: Stimulatory effect of icariin on the proliferation of neural stem cells from rat hippocampus
Source: BMC Complement Altern Med. 2018 Jan 29;18:34. doi: 10.1186/s12906-018-2095-y (PMC5789743; doi:10.1186/s12906-018-2095-y)
Supplement: Supplementary file 4 — Raw data for Fig. 6. (DOCX 19 kb) [file 12906_2018_2095_MOESM4_ESM.docx]

**Table S4.** Raw data for Fig. 6.

**The** **quantitation of cyclin D1 protein level**

| Group | β-actin | cyclin D1 | cyclin D1/ β-actin |
| --- | --- | --- | --- |
| Control 1 | 23.4 | 5 | 0.21 |
| ICA 50 μM 1 | 24.1 | 10.4 | 0.43 |
| ICA 100 μM 1 | 25.1 | 12 | 0.48 |
| Control 2 | 23.4 | 6.8 | 0.29 |
| ICA 50 μM 2 | 24.1 | 11 | 0.46 |
| ICA 100 μM 2 | 23.6 | 11.1 | 0.47 |
| Control 3 | 22.7 | 8.1 | 0.36 |
| ICA 50 μM 3 | 21.4 | 12.4 | 0.58 |
| ICA 100 μM 3 | 22.2 | 13.2 | 0.59 |
| Control 4 | 24.7 | 7.4 | 0.30 |
| ICA 50 μM 4 | 23.3 | 10.5 | 0.45 |
| ICA 100 μM 4 | 23.5 | 10.6 | 0.45 |
| Control 5 | 22.6 | 6.9 | 0.31 |
| ICA 50 μM 5 | 22 | 11.3 | 0.51 |
| ICA 100 μM 5 | 22.3 | 14.9 | 0.67 |
